# Diversity and distribution of helminth communities of the rodent *Akodon montensis* Thomas, 1913 (Rodentia: Cricetidae: Sigmodontinae) in preserved and altered environments in the Atlantic Forest, Brazil

**DOI:** 10.1016/j.ijppaw.2025.101106

**Published:** 2025-06-19

**Authors:** Camila dos Santos Lucio, Thiago dos Santos Cardoso, Rosana Gentile, Viviane Brito Dias, Milton Cezar Ribeiro, Bernardo Rodrigues Teixeira, Paulo Sérgio D'Andrea

**Affiliations:** a Graduate Program in Parasitic Biology, Oswaldo Cruz Institute/Fiocruz, RJ, Brazil; b Laboratory of Biology and Parasitology of Wild Mammal Reservoirs, Oswaldo Cruz Institute/Fiocruz, RJ, Brazil; c Graduate Program in Biodiversity, São Paulo State University (UNESP), São José do Rio Preto, SP, Brazil; d Spatial Ecology and Conservation Lab (LEEC), São Paulo State University (UNESP), Rio Claro, SP, Brazil; e Environmental Studies Center (CEA), São Paulo State University (UNESP), Rio Claro, SP, Brazil

**Keywords:** Ecology, Parasitism, Host-parasite interactions, Small mammals

## Abstract

The conversion of natural landscapes into agricultural and urban areas results in vegetation loss and habitat fragmentation, affecting biodiversity and favoring generalist species, such as *Akodon montensis*. This rodent species acts as a reservoir for zoonotic agents and harbors various parasites, including helminths, which are important indicators of environmental changes. The present study analyzed the structure, composition, and diversity of the helminth community of *A. montensis* in the Atlantic Forest, considering four locations in the state of São Paulo, Brazil. We calculated parasitological parameters (abundance, intensity and prevalence of infection) for each helminth species and evaluated helminth diversity within and between each individual host. In addition, we assessed the influence of type of environment (preserved forest or altered matrix) and host characteristics (sex and body size) on parasitological parameters of helminth species and on helminth diversity. A total of 64 individuals of *A. montensis* were analyzed, of which 60.9 % were infected with at least one helminth species. Nine species were collected, including eight nematodes and one cestode. No influence of the type of environment and host characteristics was observed on the parasitological parameters of some helminth species (e.g., *Protospirura numidica criceticola*, *Rodentolepis akodontis*, *Stilestrongylus eta* and *Syphacia* (*Syphacia*) *carlitosi*) and on the diversity of helminths within each individual host. A low effect of the type of environment on the diversity of parasites was observed among infracommunities. As an opportunistic species, *A. montensis* can exploit a wide range of resources and environmental conditions, making it less susceptible to variations in parasite diversity across habitats. These findings reinforce the importance of investigating parasite fauna in different landscapes to understand the impacts of anthropization on host-parasite interactions.

## Highlights

• Helmint communities of *Akodon montensis* were analyzed in forest and altered habitats.
• Local helminth diversity did not vary significantly between environments.
• Environment had little effect on diversity among infracommunities.
• *Akodon montensis* ecological plasticity may promote homogeneity in parasite exposure.

## Graphical abstract

**Figure:**
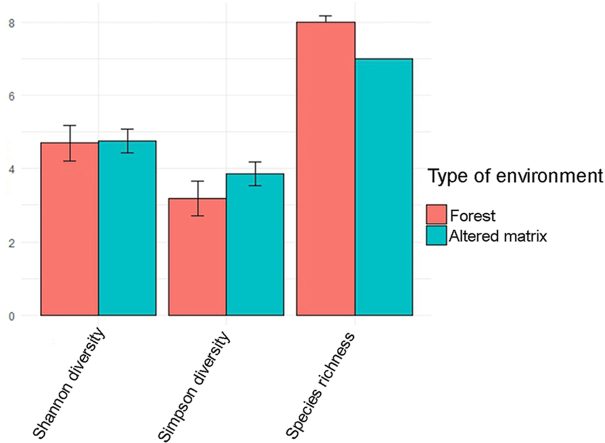

## 1 Introduction

The conversion of natural landscapes into agricultural and/or urban areas has led to vegetation loss and habitat fragmentation, thereby creating edge effects and reducing landscape connectivity (Gimenes and Anjos, 2003). These factors pose severe threats to biodiversity, compromising ecosystem health and services, as well as species survival (Meireles et al., 2022). Additionally, they alter ecological interactions and modify the composition and structure of biological communities (Fahrig, 2003; Haddad et al., 2015). Parasites are doubly affected by fragmentation, as they not only rely directly on the presence of suitable hosts, but their diversity is also strongly linked to host species richness and abundance (Dobson et al., 2008). Consequently, the loss of host species due to landscape modification can lead to secondary parasite extinctions, particularly for those with complex or host-specific life cycles (Lafferty and Kuris, 2002). However, the effects of habitat loss, fragmentation, and habitat type on parasite fauna remain poorly understood, especially in the Atlantic Forest (Bitters et al., 2022)—a recognized biodiversity hotspot.

The Atlantic Forest is an example of a Brazilian biome that has suffered intense loss of vegetation (Rezende et al., 2018; Vancine et al. 2024), with 24 % of forest remnants, of which 12.4 % are well-preserved forests, and the rest are small isolated fragments forming vegetation mosaics (Ribeiro et al., 2009; Pinheiro and Pinho, 2013; SOS Mata Atlântica Foundation, 2018). Anthropogenic alterations in this biome may favor habitat generalist species (Pardini et al., 2010), such as the terrestrial insectivorous-omnivorous rodent *A*. *montensis* (Graipel, 2003; Paglia et al., 2012). This species, which is common in disturbed areas (Bonvincino et al., 2008; D’Elía and Pardiñas, 2015), harbours various parasites and acts as a natural reservoir of zoonotic agents, such as orthohantaviruses (Oliveira et al., 2014), *Rickettsia* spp. (Rozental et al., 2017), *Trypanosoma cruzi* (Jansen et al., 2015), and helminths (Mills, 2006), which have impact on human and animal health.

Helminths are among the main groups of parasites associated with rodent hosts (Han et al., 2016; Kersul et al., 2020) and serve as important indicators of environmental change (Vidal-Martínez and Wunderlich, 2017). Interactions between helminths and wild small mammals can reveal how environmental factors influence parasitism and how habitat quality affects the distribution and occurrence of both hosts and parasites (Behnke et al., 1999; Cardoso et al., 2016). Helminth diversity in small mammals tends to be higher in well-preserved environments, where ecological conditions support a greater variety of parasite life cycles (Bordes and Morand, 2008; Cardoso et al., 2020). In such habitats, the complexity of food webs, stable microclimates, and higher host biodiversity favor the maintenance and transmission of parasites, particularly those with indirect life cycles that rely on multiple hosts or environmental stages (Lafferty and Kuris, 1999; Kersul et al., 2020). In contrast, anthropogenically altered environments often lead to the decline of specialist species and disrupt parasite transmission dynamics, resulting in reduced helminth diversity and simplified community structures (Lafferty and Kuris, 1999; Vidal-Martínez and Wunderlich, 2017; Cardoso et al., 2016).

The understanding of host–parasite relationships in fragmented landscapes can be enhanced by incorporating the concept of “*ecological edges”*. Warburton and Blanar (2021)proposed a conceptual framework that distinguishes between two types of edges: *soft edges*, which act as permeable corridors that facilitate host movement and promote parasite transmission, and *hard edges*, which function as ecological barriers, limiting dispersal and increasing the risk of local parasite extinction. Parasites with simple life cycles and direct transmission modes tend to benefit from host aggregation in soft edges, whereas those with complex life cycles or high host specificity are more negatively affected in hard edges.

In addition, the variation in parasite richness and abundance among host species is related to ecological traits of the hosts, such as population density, body size, longevity, and reproductive behavior (Arneberg et al., 1998). For instance, host species with greater body mass may support a higher diversity of parasites due to the increased availability of ecological niches (Ezenwa et al., 2006), and host population density also plays a crucial role in shaping parasitism dynamics, since host species occurring at high densities are more likely to be encountered by parasites in nature than host species with low population densities (Kamiya et al., 2014).

Understanding the factors that affect parasite transmission dynamics in natural host populations is critical for comprehending the relationships among human, animal, and environmental health (Jenkins et al., 2015; Lainé and Morand, 2020) and for biodiversity conservation (Andreazzi et al., 2023; Costa et al., 2024). This study aimed to describe the structure, composition, and diversity of the helminth community of *A. montensis* in the Atlantic Forest, encompassing both forest and altered matrix environments across four localities in São Paulo state (SP), Brazil. We analyzed parasite community diversity at the infracommunity level, which consider each host as a local community (Bush et al., 1997) and among infracommunities. Additionally, we analyzed the influence of the type of environment (forest r altered matrix) and host characteristics (sex and body size) on the parasitological parameters (abundance, intensity and prevalence) of helminth species and on parasite diversity indices. We hypothesized that hosts from forest environments would exhibit higher parasite diversity compared to those from altered matrices, given that habitat degradation may reduce helminth species richness and abundance by limiting the conditions necessary for their life cycle development (Lafferty and Kuris, 1999).

## 2 Materials and methods

### 2.1 Study area

**Fig. 1:**
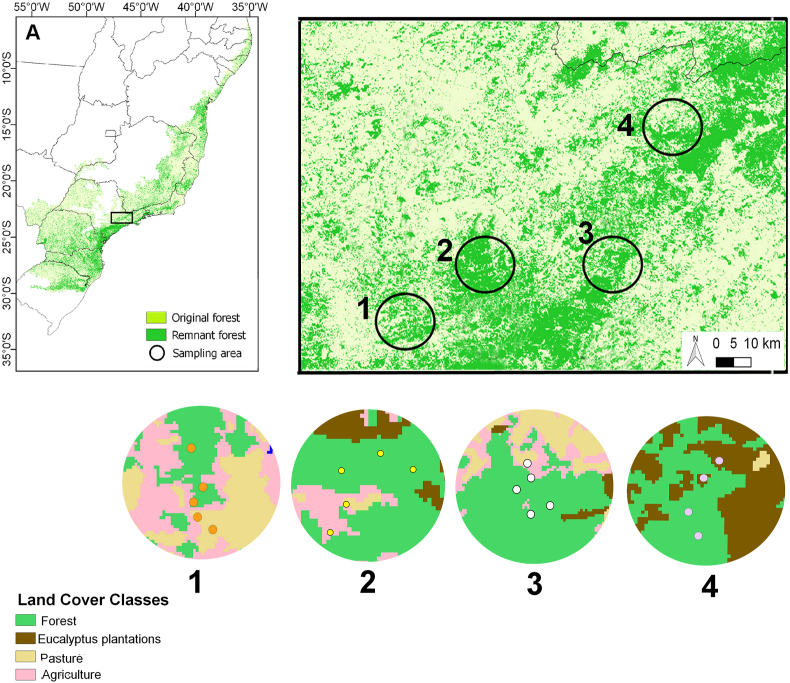
Sampling landscapes with a 5 km radius within the Cantareira-Mantiqueira Corridor, located in the state of São Paulo, Brazil. (A) Brazil, showing the original Atlantic Forest areas and the remaining fragments. (B) Location of the four sampling areas within the study region. (C) Land cover classes of the sampled landscapes (1- Atibaia, 2- Jarinú, 3- Piracaia and 4- Joanópolis).

Four localities (Atibaia-SP, Piracaia-SP, Jarinú-SP, and Joanópolis-SP) were selected within the Long-Term Ecological Research Program Cantareira-Mantiqueira Corridor (LTER CCM or PELD-CCM in Portuguese) – in the northeastern portion of São Paulo state and southern Minas Gerais state, within the Atlantic Forest biome. Most part of the vegetation of the Cantareira-Mantiqueira Corridor consists of ombrophilous mixed forests that regenerated after the intense agricultural cycles of coffee and tea dating back to the late 19th century (Fasiaben et al., 2015). The forested areas are surrounded by an anthropogenic landscape, which consists on agricultural and pasture matrices, urban areas and eucalyptus plantations (Fig. 1). This region represents an important remnant of Atlantic Forest, which originally covered more than 1.4 million km^2^ of the Brazilian territory (Silva and Casteleti, 2003). Currently, however, less than 30 % of the original forest remains in highly isolated fragments with pronounced edge effects in this region (Ribeiro et al., 2009; Joly et al., 2014; Rezende et al., 2018; Vancine et al. 2024).

### 2.2 Collection and examination of rodents

The captures of *A. montensis* were carried out in the four localities cited above. In each locality, four sites (in Joanópolis) or five sites (in Atibaia, Jarinú and Piracaia) were installed within a radius of 1000 m, at least 200 m apart. Each site had a squared grid of 25 trapping points (5 × 5) with two Sherman® live trap (Model XLK, 3 in × 3.75 in × 12in, Florida, USA) placed on the ground per point. In Joanópolis, there were two sites at the forest environment and two at the altered matrix, and in Atibaia, Jarinú and Piracaia, there were three sites at the forest environment and two at the altered matrix, totaling eleven sites at the forest environment and eight in the altered matrix.

The traps were baited with a mixture of banana, cornmeal, sardine, or vanilla essence, which is suitable for capturing small mammals with various dietary habits (granivores, frugivores, carnivores-insectivores, and omnivores). Sampling was conducted during five consecutive nights in March 2023 (Atibaia), August 2023 (Jarinú), and October 2023 (Joanópolis and Piracaia). The rodent *A. montensis* and its helminths were used as the target host-parasite interaction in this study, as this host species was present in all sampled locations, presenting high abundance among the different types of environment studied. These rodents were weighted, measured, had their sex and trapping point recorded and euthanized to collect helminths and other biological samples for zoonotic agent diagnosis of associated projects. Specimens were submitted to taxidermy and deposited in the mammal collection at the Luiz de Queiroz College of Agriculture, University of São Paulo (USP) in Piracicaba city. Rodent identification was performed based on the external morphology, cranial morphometry, and molecular sequencing. Thoracic and abdominal cavities were examined in the field, and each digestive system was stored separately (stomach, small intestine, and large intestine) in 50 ml Falcon tubes preserved in 70 % ethanol for later helminth analysis in the laboratory.

All field procedures followed the standards of capture, handling, and care recommended by the Ethics Committee on the Use of Animals of São Paulo State University (UNESP/Jaboticabal) (license CEUA nº586/22) and a federal license for the capture of wild mammals (87257–1 and 80901-2 MMA/ICMBio/SISBIO).

### 2.3 Collection and identification of helminths

The thoracic and abdominal cavities of the rodent specimens, including the stomach and small and large intestines, were examined under a stereoscopic microscope for helminth identification. All helminths were washed twice in saline to remove tissue debris and preserved in 70 % ethanol for later identification and counting. Nematodes were cleared with lactophenol, and cestodes were stained with Langeron's carmine, differentiated with 0.5 % hydrochloric acid, dehydrated in an alcohol series, diaphanized in methyl salicylate, and mounted in Canada balsam on microscopic slides (Amato et al., 1991, modified). For some trichostrongylid species, cross-sections were obtained to examine the synlophe using a stereoscopic microscope. the specimens were analyzed by light microscopy, and morphometry was conducted using digital images captured with a Zeiss AxioCam HRC (Göttingen, Germany) and AxioVision Rel. 4.7 software (Zeiss, Göttingen, Germany). Taxonomic identification of helminths was based on Travassos (1937), Yamaguti (1961), Khalil et al. (1994), Vicente et al. (1997), and Anderson et al. (2009). The specimens were deposited in the scientific helminth collection of the Laboratory of Biology and Parasitology of Wild Mammal Reservoirs, IOC/Fiocruz-RJ.

### 2.4 Data analysis

Parasitological parameters such as abundance, prevalence and intensity were calculated for each helminth species according to Bush et al. (1997), considering the variables host sex (male and female) and type of environment. Overall species richness was determined as the total number of species considering all localities. The mean species richness was calculated as the total number of species in each infracommunity divided by the number of analyzed infracommunities. The mean abundance was the total number of helminths of a species divided by the number of hosts analyzed. The mean intensity was the total number of helminths of a species divided by the number of infected hosts. Prevalence was the ratio of infected hosts to total analyzed hosts.

We investigated the influence of the type of environment (forest and altered matrix) and host characteristics (sex and body size) on abundance and intensity using Linear Multiple Regression and on prevalence using Logistic Multiple Regression. This analysis was performed separately for each parasite species with prevalence above 10 %. We also investigated this relationship between variables using mixed regressions, considering the localities as a random effect in the models. However, the localities did not show good adjustment performance, indicating that the variance of the random intercept attributed to the different localities was, for the most part, equal to zero (Supplementary Table S1). Hill indices (Hill, 1973) were calculated for each infracommunity based on Chao et al. (2014a) to analyse the effects of the type of environment (forest and altered matrix) and host characteristics (sex and body size) on species richness (q = 0), species diversity - Shannon-Wiener diversity (H′) (q = 1) and Simpson diversity (q = 2) using linear multiple regression. The Shannon index may highlight communities with many rare species, while the Simpson index may reveal communities dominated by a few species (Chao et al., 2014b). In addition, by including both Shannon and Simpson indices, we can mitigate the risk of drawing misleading conclusions based on a single measure (Chao et al., 2014b), thus enhancing the robustness of our findings. This approach allows us to assess not only how many parasite species there are, but how they are distributed in terms of abundance among hosts and type of environment.

Rarefaction/extrapolation curves were also developed based on Hill's diversity indices, in order to compare the diversity between the two types of environments. The influence of the type of environment and host sex on the similarity of the helminth diversity between infracommunities was evaluated using a Permutational Multivariate Analysis of Variance (PERMANOVA) of distance matrices. To perform this analysis, a dissimilarity matrix of helminth abundances between each pair of infracommunity was constructed using the Bray-Curtis distance index (Legendre, 2014). To visualize this result, a Principal Coordinates Analysis (PCoA) was performed, based on the Bray-Curtis dissimilarity matrix, using confidence ellipses to represent the distribution of the groups (type of environment and host sex) in the multivariate space.

The Hill numbers and rarefaction/extrapolation curves were calculated using the iNEXT package (Chao et al., 2014b, Chao et al., 2014a), the linear regression, the distance matrix and the PERMANOVA analyses were performed using the vegan package (Oksanen et al., 2024), and PCoA analysis was performed in the stats package (R Core R Development Core Team, 2024), all in R software version 4.4.2 ( R Development Core Team, 2024). The significance level considered was 0.05 in all the analyses.

## 3 results

### 3.1 Helminth fauna and parasite diversity

Sixty-four specimens of *A. montensis* were collected and analyzed, among which 60.9 % (n = 39) were infected with at least one helminth species. A total of 707 helminth specimens were collected. Nine helminth species were identified (Table 1): eight nematodes – *Protospirura numidica criceticola* (Quentin, Karimi & Rodrigues de Almeida, 1968), *Stilestrongylus freitasi* (Durette-Desset, 1968), *Stilestrongylus aculeata* (Travassos, 1918), *Stilestrongylus eta* (Travassos, 1937), *Stilestrongylus lanfrediae* (Souza et al., 2009), *Trichofreitasia lenti* (Sutton and Durette-Desset, 1991) , *Syphacia* (*Syphacia*) *carlitosi* (Robles and Navone, 2007), and *Trichuris navonae* (Robles, 2011), and one cestode – *Rodentolepis akodontis* (Rêgo, 1967).

**Table 1:** Mean abundance (±SD), mean intensity (±SD), prevalence (95 % confidence intervals), and site of infection of helminth species from *Akodon montensis* (Rodentia, Cricetidae) in relation to host sex and type of environment in Atibaia, Jarinú, Joanópolis and Piracaia, state of São Paulo, Brazil. The dash (−) indicates absence of the species.

Helminth species	*P. numidica criceticola*	*S. aculeata*	*S. eta*	*S. freitasi*	*S. lanfrediae*	*T. lenti*
Indection site	Stomach	Small intestine	Small intestine	Small intestine	Small intestine	Small intestine
Life cycle	Indirect	Direct	Direct	Direct	Direct	Direct
**Abundance**	**0.42 ± 1.70**	**1.13 ± 3.31**	**2.52 ± 14.20**	**0.45 ± 4.55**	**0.80 ± 4.47**	**0.52 ± 2.23**

Female	0.52 ± 2.16	2.23 ± 4.51	4.35 ± 20.15	–	0.84 ± 4.67	0.16 ± 0.90
Male	0.33 ± 1.05	0.09 ± 0.52	0.79 ± 1.96	0.88 ± 3.98	0.76 ± 4.35	0.85 ± 2.97
Degraded matrix	0.59 ± 2.11	1.47 ± 3.58	3.97 ± 19.25	–	–	0.15 ± 0.86
Forest	0.23 ± 0.97	0.73 ± 2.98	0.87 ± 2.05	0.97 ± 4.17	1.70 ± 6.47	0.93 ± 3.11

**Intensity**	**3.00 ± 3.64**	**9.00 ± 4.21**	**17.89 ± 35.40**	**14.50 ± 10.60**	**25.50 ± 0.71**	**8.25 ± 4.43**

Female	3.20 ± 4.92	9.86 ± 3.72	33.75 ± 52.30	–	26.00 ± 0.00	5.00 ± 0.00
Male	2.75 ± 1.71	3.00 ± 0.00	5.2 ± 1.48	14.50 ± 10.60	25.00 ± 0.00	9.33 ± 4.73
Degraded matrix	2.86 ± 4.10	8.33 ± 4.03	33.75 ± 52.30	–	–	5.00 ± 0.00
Forest	3.50 ± 2.12	11.00 ± 5.65	5.20 ± 1.48	14.50 ± 10.60	25.50 ± 0.71	9.33 ± 4.73

**Prevalence**	**14.06 (12.75–15.37)**	**12.25 (9.90–15.10)**	**14.06 (2.93–25.19)**	**3.13 (-0.44–6.69)**	**3.13 (-0.38–6.63)**	**6.25 (4.50–8.00)**

Female	16.13 (13.70–18.56)	22.58 (17.50–27.66)	12.90 (−9.78–35.59)	–	3.23 (−2.03 - 8.49)	3.22 (2.21–4.24)
Male	12.12 (10.97–13.27)	3.03 (2.46–3.60)	15.15 (13.00–17.30)	6.06 (1–71 - 10.40)	3.03 (−1.72–7.78)	9.09 (5.85–12.33)
Degraded matrix	20.59 (18.32–22.85)	17.65 (13.79–21.50)	11.76 (−8.94–32.47)	–	–	2.94 2.02–3.86)
Forest	6.67 (5.56–7.78)	6.67 (3.25–10.08)	16.67 (14.32–19.01)	6.67 (1.89–11.44)	6.67 (−0.74–14.08)	10.00 (6.44–13.56)

Sy (Sy.) carlotosi	Tr. navonae	R. akodontis
Large intestine	Large intestine	*Small intestine*
Direct	Direct	Indirect
**4.50 ± 10.98**	**0.28 ± 1.16**	**0.44 ± 1.48**

2.10 ± 5.51	0.45 ± 1.50	0.42 ± 1.82
6.76 ± 14.07	0.12 ± 0.69	0.45 ± 1.03
3.06 ± 8.14	0.53 ± 1.56	0.65 ± 1.91
6.13 ± 13.47	–	0.20 ± 0.61

**18.00 ± 15.72**	**4.50 ± 1.73**	**3.11 ± 2.71**

13.00 ± 7.11	4.67 ± 2.08	4.33 ± 4.93
20.27 ± 18.22	4.00 ± 0.00	5.00 ± 0.84
17.33 ± 9.20	4.50 ± 1.73	3.67 ± 3.27
16,72 ± 19,19	–	2.00 ± 0.00

**25.00 (16.40–33.61)**	**6.25 (5.34–7.16)**	**14.06 (12.90–15.22)**

16.13 (9.92–22.33)	9.68 (7.99–11.37)	9.68 (7.62–11.73)
33.33 (17.97–48.70)	3.03 (2.27–3.79)	18.18 (17.05–19.31)
17.65 (8.90–26.40)	11.76 (10.08–13.44)	17.65 (15.60–19.70)
36,67 (21,25–52,08)	–	10.00 (9.30–10.70)

The nematodes *P. numidica criceticola* (14.06 %), *S. aculeata* (12.25 %), *S. eta* (14.06 %), *Sy.* (*Sy.) carlitosi* (25.00 %), and the cestode *R. akodontis* (14.06 %) were the most prevalent, while the nematodes *Sy*. (*Sy.) carlitosi* (4.50 ± 10.98), *S. eta* (2.52 ± 14.20)and *S. aculeata* (1.13 ± 3.31) were the most abundant (Table 1).

No statistically significant effect of the type of environment and host characteristics (sex and body size) was recorded on the parasitological parameters of *P. numidica criceticola*, *R. akodontis*, *S. eta* and *Sy*. (*Sy.) carlitosi* (Table 2).

**Table 2:** Regression values between parasitological descriptors of the helminths from *Akodon montensis* and explanatory variables: type of environment (altered matrix or forest), host sex (male or female), and host body size. R^2^ = proportion of total variance explained by the regression model (model goodness-of-fit); F = ratio of variance between sample means to variance within samples; Deviance = measure of model fit based on likelihood comparisons; D.F. = degrees of freedom; P-value = significance value, considering α ⩽ 0.05. Only helminth species with prevalence above 10 % were analyzed.

Parasitological descriptor	Helminth species	R^2^	F or Deviance	DF	P
Abundance					
	*Protospirura numidica criceticola*	4.19 × 10−4	1.01	3	0.4
	*Rodentolepis akodontis*	−0.02	0.77	3	0.52
	*Stilestrongylus eta*	−0.02	0.7	3	0.56
	*Syphacia carlitosi*	−0.03	0.62	3	0.61

Intensity					
	*Protospirura numidica criceticola*	0.46	3.23	3	0.12
	*Rodentolepis akodontis*	−0.13	0.69	3	0.59
	*Stilestrongylus eta*	0.17	1.63	3	0.28
	*Syphacia carlitosi*	−0.13	0.43	3	0.73

Prevalence					
	*Protospirura numidica criceticola*	0.11	4.82	3	0.19
	*Rodentolepis akodontis*	0.08	3.3	3	0.35
	*Stilestrongylus eta*	0.09	3.99	3	0.26
	*Syphacia carlitosi*	0.07	3.62	3	0.310

**Fig. 2:**
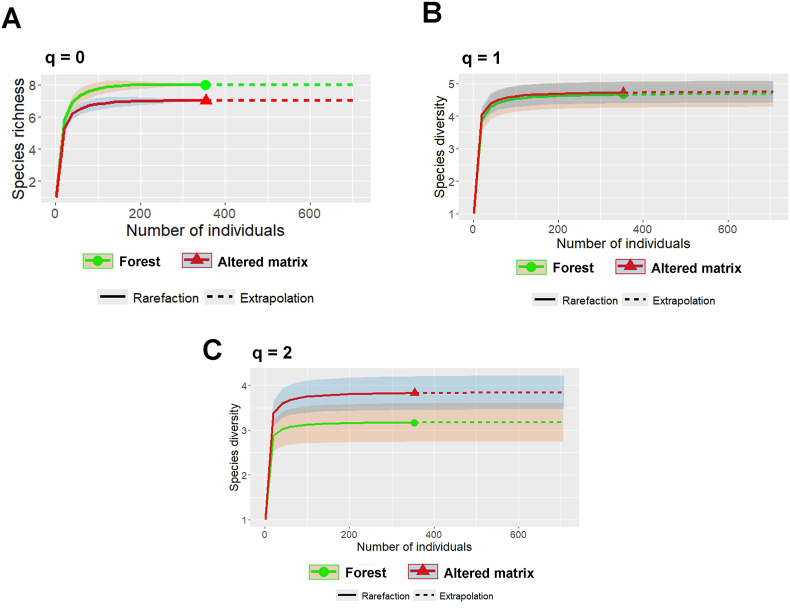
Rarefaction/Extrapolation Curves of Hill Numbers for helminths from *Akodon montensis* in relation to the type of environment (Forest or Altered matrix). A (q = 0): Richness-based diversity; B (q = 1): Shannon diversity, and; C (q = 2): Simpson diversity.

Observed and extrapolated helminth richness and diversity values were similar when comparing the forest environment to the altered matrix environment (Fig. 2A, B and 2C). In fact, no effect of the type of environment and host characteristics was observed on the Hill diversity indices (Table 3).

**Table 3:** Regression values between Hill's diversity indices for the *Akodon montensis* helminth community and the type of enviroment (altered matrix or forest), host sex (male or female) and host body size.

Hill diversity	Coefficients			
	R^2^	F	DF	P-value
Species richnnes	−0.02	0.78	3	0.51
Shannon	−0.02	0.80	3	0.50
Simpson	5.71 × 10^−4^	1.01	3	0.40

**Fig. 3:**
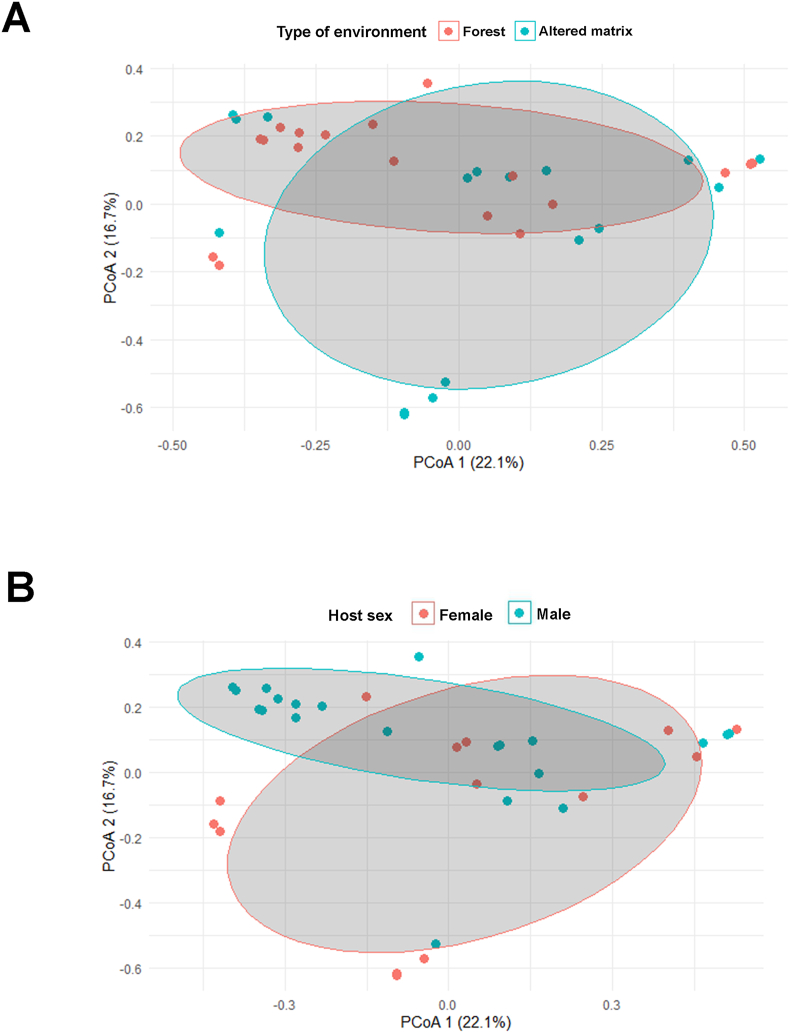
Plot of helminth diversity values between host specimens (betadiversity) of *Akodon montensis* in relation to the (A) type of environment (Forest or Altered matrix) and (B) host sex (Female or Male).

Corroborating the regressions, the results of the PERMANOVA indicated a weak relationship between the type of environment and the helminth diversity among infracommunities (Df = 1; R^2^ = 0.05; F = 2.02; p value = 0.04) (Fig. 3A). In addition, no significant relationship was observed between host sex and helminth diversity among infracommunities (DF = 1, R2 = 0.05; F = 1.80; p-value = 0.07) (Fig. 3B).

## 4 Discussion

### 4.1 Helminth fauna and parasite diversity

Considering the helminth fauna, of the nine species found in the present study, eight had been previously reported parasitizing *A. montensis*. The scientific literature includes several studies on the helminth fauna of the genus *Akodon* (Cardoso et al., 2019; Boullosa et al., 2020; Benatti et al., 2021; Gomes et al., 2003; Panisse et al., 2017; Simões et al., 2011; Kersul et al., 2020), however, few of these have been conducted in anthropogenically altered environments. (Cardoso et al., 2016; Kersul et al., 2020; Lucio et al. 2021; Gentile et al., 2022). Lucio et al. (2021) reported the single study of the helminth fauna of *Akodon cursor* in altered matrix environments in the Atlantic Forest, in which eight helminth species were recorded. The richness of the helminth fauna reported in this study (nine species) for *A. montensis* was similar to that described in the literature for this host in preserved forest areas and forest fragments in other regions of the Atlantic Forest, varying from six to 11 species as following. Six helminth species have been reported in preserved environments in Petrópolis, Rio de Janeiro State (Cardoso et al., 2016) and Santa Catarina State (Boullosa et al., 2020), seven species in forest fragments in São Paulo state (Püttker et al., 2008), eight species in Misiones Province, Argentina (Panisse et al., 2017), 10 species in forest fragments in Paraná state (Benatti et al., 2021), and 11 species in forest fragments and preserved forests in Teresópolis, Rio de Janeiro state (Simões et al., 2011).

Considering both types of environments, two helminth species were exclusive to forest areas (*S. eta* and *S. lanfrediae*), while one species was recorded only in the altered matrix environment (*Tr. navonae*). However, no significant differences were found in helminth infracommunities between the environments. This pattern may be explained by the interaction between ecological and behavioral traits of the host species and biological characteristics of the parasites involved. *Akodon montensis* is widely recognized as a generalist and opportunistic species, with a broad distribution across South America and a high tolerance to anthropogenic environments (Pardini et al., 2010; Bonvincino et al., 2008) and altered landscape matrices (Furtado et al., 2013). This ecological plasticity in diet and habitat use, combined with functional connectivity between forest fragments and altered areas for *A. montensis*, may facilitate the movement of individuals and the circulation of infective stages in the environment (Mendes et al., 2022; Wells et al., 2007). Consequently, this may promote a homogeneous exposure to helminth infective forms regardless of vegetation type (Panisse et al., 2017; Benatti et al., 2021), thus reducing the expected differences in helminth communities between forest and altered environments (Püttker et al., 2008; Lafferty, 1997)).

Similarly, no differences were detected in helminth infracommunities between males and females of *A. montensis*. This pattern suggests that both sexes share similar strategies of space use, trophic behavior and movement, which translates into equivalent levels of exposure to the infective forms (Cardoso et al., 2018; Simões et al., 2011). Nevertheless, several studies of helminths of wild rodents reported parasitological differences between sexes, often attributed to a greater movement of males, territoriality, competition for mates and immunomodulatory effects of androgens (Klein, 2004; Zuk and McKean, 1996).

Regarding the prevalence, the nematode *P. numidica criceticola* and the cestode *R. Akodontis*, which have indirect life cycles requiring intermediate arthropod hosts, were noteworthy. *Protospirura numidica criceticola* belongs to the order Spirurida, which includes several superfamilies that use insects, such as crickets, grasshoppers, cockroaches and beetles, as intermediate hosts (Gray and Anderson, 1982). Studies by Cawthorn and Anderson (1976) reported the development of *Physaloptera maxillaris*, also of the order Spirurida, in wild crickets. For *R. Akodontis*, of the order Cyclophyllidea, research indicates that intermediate hosts such as beetles, fleas, and cockroaches play significant roles in the life cycles of species in this order, such as *Hymenolepis nana* (Heicher and Gallati, 1978). In this study, the mean abundance and prevalence of *P. numidica criceticola* and *R. Akodontis* were greater than those reported in other studies in the Atlantic Forest (Simões et al., 2011; Cardoso et al., 2016; Boullosa et al., 2020; Benatti et al., 2021).

The lack of a clear effect of habitat type or host characteristics on helminth diversity at the infracommunity level suggests a relatively uniform distribution of factors influencing parasite diversity among hosts. This pattern may reflect the ecological plasticity of *A. montensis*, a generalist species capable of exploiting varied resources across different environments, which may facilitate exposure to a broad range of parasites. Additionally, many of the helminth species found may be well adapted to both preserved and altered environments, contributing to similar parasite assemblages across habitats. Nonetheless, unmeasured variables—such as individual dietary differences or variation in immune response— may also modulate parasite acquisition and help explain observed patterns of diversity (Behnke et al., 1999; Dallas et al., 2020).

## CRediT authorship contribution statement

**Camila dos Santos Lucio:** Writing – review & editing, Writing – original draft, Visualization, Validation, Software, Resources, Methodology, Investigation, Funding acquisition, Formal analysis, Data curation, Conceptualization. **Thiago dos Santos Cardoso:** Writing – review & editing, Writing – original draft, Software, Resources, Methodology, Investigation, Formal analysis, Conceptualization. **Rosana Gentile:** Writing – review & editing, Writing – original draft, Methodology, Formal analysis. **Viviane Brito Dias:** Writing – review & editing, Methodology, Investigation, Data curation. **Milton Cezar Ribeiro:** Writing – review & editing, Project administration, Methodology, Investigation, Funding acquisition. **Bernardo Rodrigues Teixeira:** Writing – review & editing, Writing – original draft, Funding acquisition, Formal analysis. **Paulo Sérgio D'Andrea:** Writing – review & editing, Writing – original draft, Resources, Methodology, Investigation, Funding acquisition, Data curation, Conceptualization.

## 5 Funding

CSL received grants from Oswaldo Cruz Institute IOC/Fiocruz, the Graduate Program in Parasite Biology of the Oswaldo Cruz Institute (PGBP/IOC/Fiocruz), and Coordenação de Aperfeiçoamento de Pessoal de Nível Superior - Brazil (CAPES). TSC received grants from Fundação Carlos Chagas Filho de Amparo à Pesquisa do Estado do Rio de Janeiro-FAPERJ, process number E−26/204.420/2021. MCR thanks the São Paulo Research Foundation - FAPESP (processes #2013/50421–2; #2020/01779–5; #2021/06668–0; #2021/08322–3; #2021/08534–0; #2021/10195–0; #2021/10639–5; #2022/10760–1) and the National Council for Scientific and Technological Development - CNPq (processes #442147/2020–1; #402765/2021–4; #313016/2021–6; #440145/2022–8; 420094/2023–7; 446029/2024–6; 421464/2025–9) and São Paulo State University - UNESP for their financial support. BRT thanks the FAPERJ, process numbers E−26/211.249/2019 and E−26/211.615/2021, and CNPq process 421866/2023–3). PSD received grants from FAPERJ, process numbers E−26/210.245/2018, E−26/210.467/2019, E−26/210.309/2021 and E−26/201.223/2022 and CNPq processes 315677/2021–0. This study is also part of the *Center for Research on Biodiversity Dynamics and Climate Change*, which is financed by the São Paulo Research Foundation - FAPESP.

## Conflict of interest

The authors declare no conflict of interest.
