# Supplementary material for: Diversity and distribution of helminth communities of the rodent Akodon montensis Thomas, 1913 (Rodentia: Cricetidae: Sigmodontinae) in preserved and altered environments in the Atlantic Forest, Brazil
Source: Int J Parasitol Parasites Wildl. 2025 Jun 19;27:101106. doi: 10.1016/j.ijppaw.2025.101106 (PMC12226378; doi:10.1016/j.ijppaw.2025.101106)
Supplement: Multimedia component 1 [file mmc1.docx]

| **Helminth species** | **Parasitological parameters** | **ramdom effect (localities)** | **Significant predictor(s)** |
| --- | --- | --- | --- |
| *Protospirura numidica criceticola* | Abundance | 0 | None |
|  | Prevalence | 0 | None |
|  | Intensity | 0 | Body size (p < 0.05, t = 3.053) |
|  |  |  |  |
| *Rodentolepis akodontis* | Abundance | 0.1164 | None |
|  | Prevalence | 0.000 (≈2.34e-16) | None |
|  | Intensity | 0.8657 | None |
|  |  |  |  |
| *Stilestrongylus eta* | Abundance | 0 | None |
|  | Prevalence | 0 | None |
|  | Intensity | 912.7 | None |
|  |  |  |  |
| *Syphacia carlitosi* | Abundance | 0 | None |
|  | Prevalence | 0 | None |
|  | Intensity | 0 | None |

Table S1. Mixed regressions with values ​​of the estimated variance of the random effect (locations: Atibaia, Jarinú, Joanópolis and Piracaia) and fixed predictors (host sex, host body size and type of environment) for each helminth species and for each of the parasitological descriptors evaluated (abundance, prevalence and intensity) in rodents collected in the state of São Paulo, Brazil.
